# Spasm of the Œsophagus

**Published:** 1881-10

**Authors:** 


### SPASM OF THE ŒSOPHAGUS.

The following case of œsophageal spasm is narrated in the *Lancet*, by Dr. Moorhead :—

On April 26th, at 9:30 P. M., I was summoned to attend a gentleman, over sixty years of age, who had been suddenly seized with obstruction of the œsophagus, which completely barred the passage of any food, solid or liquid, into the stomach. On my arrival, I was informed by my patient, who seemed in excellent health, that about 8 P. M., at the close of dinner, he inadvertently swallowed a piece of meat imperfectly masticated, which did not enter the stomach, but lodged in the œsophagus near its cardiac extremity. This was recognized by the sense of oppression which immediately ensued in the lower dorsal region. A glass of sherry was at once swallowed, in the hope of facilitating the removal of the obstruction, but it was of no avail. Then some water was taken which was not only ineffectual, but immediately developed hiccough, terminating in speedy rejection of all the liquid imbibed.

With regard to treatment, I first used the probang, pushing it down to its utmost extent, but without any benefit. I then employed a bougie of the largest size, which I believe effected an entrance into the stomach after encountering some resistance near its cardiac orifice. When the instrument was withdrawn, however, and liquid swallowed, there was no passage, as the fluid was brought up in a few moments. The conclusion arrived at, therefore, was, that though the foreign body was removed, the œsophagus was completely closed by spasm; or, in other words, that the case was then one of spasmodic stricture of that tube. Perfect quietude was enjoyed, while a small draught containing thirty minims of laudanum was prescribed.

April 27th. At my visit at 7 A. M., I found that the opiate had been rejected, and that there was total obstruction. After another unsuccessful trial of the probang and bougie, I administered a quarter of a grain of morphia hypodermically, applied a warm bran poultice to the lower dorsal region, and advised small pieces of ice to be swallowed frequently.

It was then decided to discontinue further surgical interference, and to pursue only medical treatment. With this in view the liquid extract of belladonna was brushed freely over the spine, in the dorsal region, while ten minims of the tincture of the same drug was administered in teaspoonful doses ever four hours. This treatment was continued until about eight o'clock in the evening, when, on swallowing several small pieces of ice in rapid succession, it was found they were no longer rejected, and that some ice cream freely entered the stomach. Shortly afterward the patient was able to regale himself with half a pint of soup. From this time the dysphagia entirely ceased, leaving only a little mucous irritation of the canal, which, however, rapidly subsided.—*Medical and Surgical Reporter.*

